# The structural dimensions of adherence to ethnic traditional sports exercise prescription and the development of its scale

**DOI:** 10.3389/fpsyg.2025.1555765

**Published:** 2025-07-01

**Authors:** Jiali He, Zuosheng Lu, Jinyong Han

**Affiliations:** ^1^School of Physical Education and Sports Science, South China Normal University, Guangzhou, China; ^2^School of Sports Science, Lingnan Normal University, Zhanjiang, China

**Keywords:** traditional sports, exercise prescription, adherence, scale development, FITT-VP principles

## Abstract

**Introduction:**

The aim of the present study was to develop and validate a multidimensional adherence scale for ethnic traditional sports exercise prescriptions, specifically tailored for older adults in China. The scale was grounded in Social Cognitive Theory and was guided by the FITT-VP principles (Frequency, Intensity, Time, Type, Volume, and Progression).

**Methods:**

The sample comprised 342 retired older adults (aged 55–80 years), with a mean age of 67.5 years (SD = 6.3), recruited from 10 universities in Guangdong Province. To verify the psychometric properties of the scale, exploratory factor analysis (EFA) and confirmatory factor analysis (CFA) were performed to determine the factor structure and model fit.

**Results:**

Results showed that the finalized 23-item scale presented a three-factor solution—Cognitive Adherence, Behavioral Adherence, and Self-Regulatory Adherence—accounting for 71.7% of the total variance. CFA supported the three-factor model (χ^2^/df = 4.70, RMSEA = 0.10), with satisfactory factor loadings (≥ 0.50) and acceptable fit indices (NFI = 0.87, CFI = 0.88, RMR = 0.05, GFI = 0.80). Internal consistency, measured by Cronbach’s *α*, ranged from 0.92 to 0.96 for the subscales and achieved 0.97 for the overall scale. Criterion-related validity analysis indicated significant positive correlations (*p* < 0.01) with an established adherence scale, supporting the external validity of the new scale. Construct validity, tested through convergent and discriminant validity measures, supported the use of the scale in evaluating adherence to ethnic traditional sports exercise prescriptions.

**Discussion:**

These findings highlight the need for culturally tailored assessment tools and the importance of addressing cognitive, behavioral, and self-regulatory dimensions to enhance adherence. Despite these strengths, the study has limitations, including the relatively homogeneous sample of retired university faculty from a single province, which may limit generalizability to more diverse elderly populations. As a general conclusion, the developed scale appears to be a valid and reliable tool for assessing adherence to ethnic traditional sports among older adults. This multidimensional tool may help professionals design more effective, theory-driven interventions, ultimately promoting active and healthy aging in culturally relevant ways.

## 1 Introduction

Regular physical activity has been widely recognized as a key factor in the prevention and management of chronic diseases (Warburton and Bredin, 2017). In recent years, ethnic traditional exercises such as Baduanjin and Tai Chi have attracted growing attention for their potential to promote health and prevent disease among older adults (Chen et al., 2006; Fetherston and Wei, 2011). These forms of exercise not only carry profound cultural heritage and enjoy broad popularity, but they also offer an effective intervention strategy in response to the escalating prevalence of chronic conditions (Yeh et al., 2004).

Despite the general scholarly consensus regarding the importance of incorporating ethnic traditional sports into exercise prescriptions, low adherence remains a major challenge during the implementation phase (Sniehotta et al., 2005). Exercise prescription adherence refers to the degree to which individuals comply with and maintain the exercise program prescribed by medical or fitness professionals, and it is crucial in determining whether the anticipated health benefits can be achieved (Rhodes et al., 2017).

Existing research has predominantly explored factors influencing exercise prescription adherence from the perspectives of individual characteristics—such as demographics, motivation, and emotional states—and interpersonal factors, including patient–provider relationships and social support (Bauman et al., 2012; Sallis et al., 2021). However, this study identifies three major research gaps that need to be addressed: First, many existing scales target specific patient populations (e.g., individuals with cancer-related fatigue), limiting their applicability to broader preventive healthcare contexts (Al, 1998; Pinto et al., 2009). Second, research focusing specifically on the adherence of older adults to ethnic traditional sports prescriptions is limited. Third, few measurement tools integrate the “Golden Six Principles” of exercise prescription (FITT-VP) with Social Cognitive Theory, making it challenging to capture the multifaceted nature of exercise adherence in older populations (Bandura, 2004).

According to Bandura’s Social Cognitive Theory, individual cognition, behavior, and self-regulation interact dynamically in shaping exercise adherence, highlighting the importance of observational learning and self-efficacy (Bandura, 2004). Guided by this theoretical perspective, the present study selected retired faculty members from several universities in Guangdong Province as participants, using Baduanjin as a representative form of ethnic traditional exercise. By constructing a new scale, this research aims to reflect the interplay of cultural elements, the FITT-VP framework, and cognitive–behavioral processes that influence exercise prescription adherence among older adults.

Based on the identified research gaps, this study aims to address the following research questions: (1) What are the structural dimensions of adherence to ethnic traditional sports exercise prescriptions among elderly populations? (2) How can a reliable and valid scale be developed to measure these dimensions in accordance with the FITT-VP principles and Social Cognitive Theory? (3) What are the psychometric properties of this newly developed scale?

## 2 Methodology

### 2.1 Research design and scale development process

This study employed a mixed-methods approach to develop and validate an adherence scale for ethnic traditional sports exercise prescriptions. The research followed a systematic three-phase process: (1) initial scale development through literature review and qualitative interviews; (2) content validation through expert consultation; and (3) psychometric evaluation through quantitative survey methods. This methodological framework ensured both theoretical validity and empirical rigor throughout the scale development process.

### 2.2 Initial item construction and content revision of the scale

The Golden Six Principles of Exercise Prescription (FITT-VP) include Frequency, Intensity, Time, Type, Volume, and Progression. The development of the adherence scale for ethnic traditional sports exercise prescriptions is mainly based on the requirements from relevant exercise prescription literature, using the Golden Six Principles of Exercise Prescription (FITT-VP) as the basis for evaluation, and referring to adherence scales for osteoporosis, breast cancer, stroke functional exercise, and several other relevant works (Ekkekakis et al., 2011). The scale initially consisted of 26 items, covering six dimensions: exercise frequency (5 items), exercise intensity (5 items), exercise time (4 items), exercise type (4 items), exercise volume (4 items), and exercise progression (4 items). After the scale was developed, five review experts were invited to score the scale based on the five content assessment criteria designed by Waltz and Bausell, (1981) (relevance, clarity, brevity, ambiguity, and cultural relevance). These experts included two master’s supervisors in the field of scale development, two doctoral supervisors in the field of ethnic traditional sports, and two deputy chief physicians in the field of general practice. The scale revision process involved conducting open-ended interviews with community-dwelling older adults aged 60 and above, who were invited to evaluate the scale’s content validity and structural coherence based on their lived experiences. Based on their suggestions, the scale items were revised again to meet the requirements of being easy to understand, “situational,” and “avoiding verbosity.” The final scale was determined to consist of 24 items, including a five-point scale covering six dimensions: frequency, intensity, time, type, volume, and progression.

Each item uses a 5-point rating scale, namely “Cannot do at all,” “Occasionally cannot do,” “Generally can do,” “Basically can do,” and “Can do completely,” with scores assigned as 1, 2, 3, 4, and 5, respectively. One item is a lie detector item and is not included in the total score. The scoring method for the items is the sum of the scores for the 24 items, with a higher total score indicating a higher level of adherence to the ethnic traditional sports exercise prescription. The theoretical range for the adherence score is 24–120 points. The adherence rate was computed using linear transformation:


$$\text{Adherence Rate}\;(\%)=\frac{\begin{array}{c} \text{Actual Score}-\text{Theoretical}\;\\ \text{Minimum Score} \end{array}}{\begin{array}{c} \text{Theoretical Maximum Score}\\ -\text{Theoretical Minimum Score} \end{array}}\times 100\%$$

or,


$$\text{Adherence Rate}\;(\%)=\frac{\text{Actual Score}-24}{120-24}\times 100\%$$

This normalization procedure converts raw scores to a percentage metric reflecting relative adherence level. Following this initial scale development, we proceeded to recruit participants for psychometric testing.

### 2.3 Participants

This study focused on retired academic administrators (former university faculty members holding managerial positions) from higher education institutions in Guangdong Province. Using cluster random sampling, we selected 10 universities in Guangdong province and randomly recruited 40 retired teachers from each institution, totaling 400 participants. After excluding invalid questionnaires, 342 valid responses remained. Participants met the following inclusion criteria: (1) aged 55 or above; (2) no severe cognitive dysfunction; and (3) physical condition suitable for moderate exercise. Demographic characteristics included: age range 55–80 years (*M* = 65, SD = 7); 55% men and 45% women; educational level primarily undergraduate and above (60%), followed by associate degree (40%); marital status predominantly married (80%), with 15% widowed and 5% divorced. Occupationally, participants were primarily retired teachers (70%), administrative staff (20%), and logistics personnel (10%). The selection of this population was based on several methodological considerations: their high acceptance of traditional sports programs like Baduanjin, educational background facilitating accurate comprehension of scale items, and sample homogeneity reducing potential confounding factors during initial scale development. While the sample size was relatively modest, our research team ensured data quality through meticulous individual data collection, accurately capturing exercise prescription adherence patterns among elderly individuals. With the participant sample established, we implemented a comprehensive data collection and analysis protocol.

### 2.4 Procedure

This study strictly followed the standard procedures of psychometrics to conduct a systematic and rigorous reliability and validity test on the collected 342 valid samples to comprehensively evaluate the psychometric properties of the adherence scale for ethnic traditional sports exercise prescriptions.

1. Content validity assessment: Content validity was assessed by combining expert review and target population interviews to ensure that the scale items could accurately and comprehensively reflect the measured construct (Polit and Beck, 2006).
2. Construct validity evaluation: In the construct validity test, this study combined exploratory factor analysis (EFA) and confirmatory factor analysis (CFA). In the EFA phase, using all 342 samples, factors were extracted using the principal component analysis method, and orthogonal rotation was performed using the varimax method, with the three-factor model determined based on the criterion of eigenvalues greater than 1 and the scree plot. In the CFA phase, 210 samples were randomly selected from the total sample, and structural equation modeling technology was employed to verify the theoretical model derived from the EFA results. The model was adjusted according to the modification indices, ultimately confirming the three-factor structure of cognition, behavior, and self-regulation, providing strong empirical support for the theoretical conceptualization of adherence to ethnic traditional sports exercise prescriptions.
3. Reliability and supplementary validity testing: For reliability assessment, this study employed Cronbach’s *α* coefficient, which is widely recognized in the field of reliability analysis, to systematically assess the internal consistency reliability of the total scale and each subscale. To further examine the convergent and discriminant validity of the scale, this study also calculated two important indicators: composite reliability (CR) and average variance extracted (AVE). Additionally, to verify the criterion-related validity of the scale, this study conducted a correlation analysis using an existing exercise prescription adherence scale for gastric cancer-related fatigue patients as an external criterion.

### 2.5 Statistical methods

All data were processed and analyzed by using SPSS 23.0 and AMOS 26.0.

## 3 Results

### 3.1 Item analysis

First, the research team used item-total correlation analysis to examine the correlation between the 24 items of the scale and the total score. The results showed that the Pearson correlation coefficients between all items and the total score were above 0.56 (*p* < 0.05), indicating a high correlation between all items and the total score. The item with the highest correlation with the total score was C2 (*r* = 0.84), and the lowest was D4 (*r* = 0.56). Second, the corrected item-total correlation (CITC) and the Cronbach’s coefficient after deleting the item (CIDC) were calculated through the reliability coefficient method to further test the quality of the items. The results showed that the CITC coefficients of the 24 items ranged from 0.56 (D4) to 0.84 (C2), all above the threshold of 0.50 (Devellis, 2016). The CIDC results indicated that even if any item was deleted, the Cronbach’s *α* coefficient of the scale remained at a high level of above 0.97, demonstrating excellent internal consistency reliability. Further comparison revealed that item D4 was relatively low in both item-total correlation and CITC values, which could have a certain negative impact on the internal consistency of the scale. To further optimize the psychometric properties of the scale, the researchers ultimately decided to remove item D4. All other items had satisfactory CITC values, CIDC values, and item-total correlations, and were retained. Through a series of item analyses, this study selected 23 items with excellent psychometric properties from the original 24 items to serve as the basis for the formal scale. Based on existing research experience, this scale performed well in terms of item quality and can be used in subsequent research.

### 3.2 Exploratory factor analysis

To further determine the factor structure of the scale, we conducted an exploratory factor analysis on the collected data from 342 samples. First, the KMO value was calculated to be 0.96, and Bartlett’s test of sphericity reached a significant level (*p* < 0.05), indicating that the data was very suitable for factor analysis (Field, 2018). Using the principal component analysis method, three factors were initially extracted based on the criterion of eigenvalues greater than 1, with a cumulative variance contribution rate of 71.7%, far above the critical value of 50%, indicating that these three factors could explain most of the variation in information. At the same time, the scree plot visually showed that the eigenvalues of the first three factors were significantly higher than those of the subsequent factors, with a gradual flattening slope, further supporting the conclusion of extracting three factors.

After preliminarily determining the number of factors, to make the factor structure more interpretable, the initial factor solution was orthogonally rotated using the maximum variance method. The rotated factor loading matrix showed that all items had loadings greater than the standard value of 0.50 on the corresponding factor, and the loadings on other factors were relatively small, indicating good discrimination between the three factors, reflecting different dimensions of adherence to ethnic traditional sports exercise prescriptions.

Based on the results of the factor analysis and in combination with the theoretical basis of the questionnaire, the three factors were named “Cognitive Adherence,” “Behavioral Adherence,” and “Self-regulatory Adherence.” Among them, “Cognitive Adherence” includes items A1, A2, A3, A4, C1, C2, C3, C4, mainly reflecting the individual’s subjective cognition and internalization of exercise prescription requirements (such as exercise frequency, time); “Behavioral Adherence” includes items B1, B2, B3, B4, E1, E2, E3, E4, mainly reflecting the individual’s actual implementation of the exercise prescription (such as exercise intensity, total volume); “Self-regulatory Adherence” includes items D1, D2, D3, F1, F2, F3, F4, mainly reflecting the individual’s self-management and motivation ability during exercise. This factor structure is highly consistent with the key elements of the FITT-VP principle in exercise prescriptions (Table 1).

**Table 1:** Rotated component matrix in exploratory factor analysis.

Principle	Item	Cognitive	Behavioral	Self-Regulatory
Frequency	A1. I can complete the three times a week of Baduanjin practice suggested by the coach.	0.80		
	A2. I can maintain the three times a week of Baduanjin practice without reducing due to weather, mood, and other factors.	0.75		
	A3. I can record the frequency of exercises per week to ensure it meets the requirements of the prescription.	0.74		
	A4. I can adhere to more than three times a week of exercise frequency recommended by the coach.	0.74		
Intensity	B1. I can judge whether I am in moderate exercise intensity according to the heart rate of 80–112 beats per minute during exercise.		0.71	
	B2. I can judge whether I am in moderate exercise intensity according to whether I can talk but not easily sing during exercise.		0.49	
	B3. I can regularly provide feedback on my feelings about exercise intensity to the coach.		0.48	
	B4. I can strictly control the exercise intensity to achieve the best health effects of Baduanjin practice.		0.54	
Exercise time	C1. I can squeeze out time to complete more than half an hour of Baduanjin practice requirements even when busy.	0.77		
	C2. I can reasonably arrange time to complete at least half an hour of Baduanjin practice each time.	0.75		
	C3. I can complete the suggested prescription of at least half an hour of Baduanjin practice each time.	0.78		
	C4. I can persist in half-hour Baduanjin practice three times a week for a long time.	0.77		
Type	D1. I can communicate with the coach in time to understand the precautions of the Baduanjin practice methods.			0.46
	D2. I can study and master the Baduanjin practice movements taught by the coach.			0.64
	D3. I can follow the Baduanjin practice video recommended by the coach with curiosity and interest.			0.72
Total volume	E1. I can carefully check whether I have reached the prescription of 150 min of weekly exercise time.		0.66	
	E2. I can monitor my heart rate changes during exercise to check whether I maintain moderate intensity exercise.		0.84	
	E3. I can monitor my heart rate to maintain moderate intensity requirements and accumulate 150 min per week.		0.76	
	E4. I can flexibly adjust the time of each exercise within a week to ensure that the total weekly exercise volume meets the requirements of the prescription.		0.68	
Progression	F1. I can adapt well to the coach’s adjustments to the difficulty of the exercise prescription every once in a while.			0.46
	F2. I can maintain exercise enthusiasm for a progressive number of arrangements.			0.64
	F3. I can expect the coach to give me a more difficult exercise prescription.			0.72
	F4. I can firmly believe that the progressive number of exercise prescriptions can continuously improve my physical condition.			0.73

### 3.3 Model fit test

**Figure 1:**
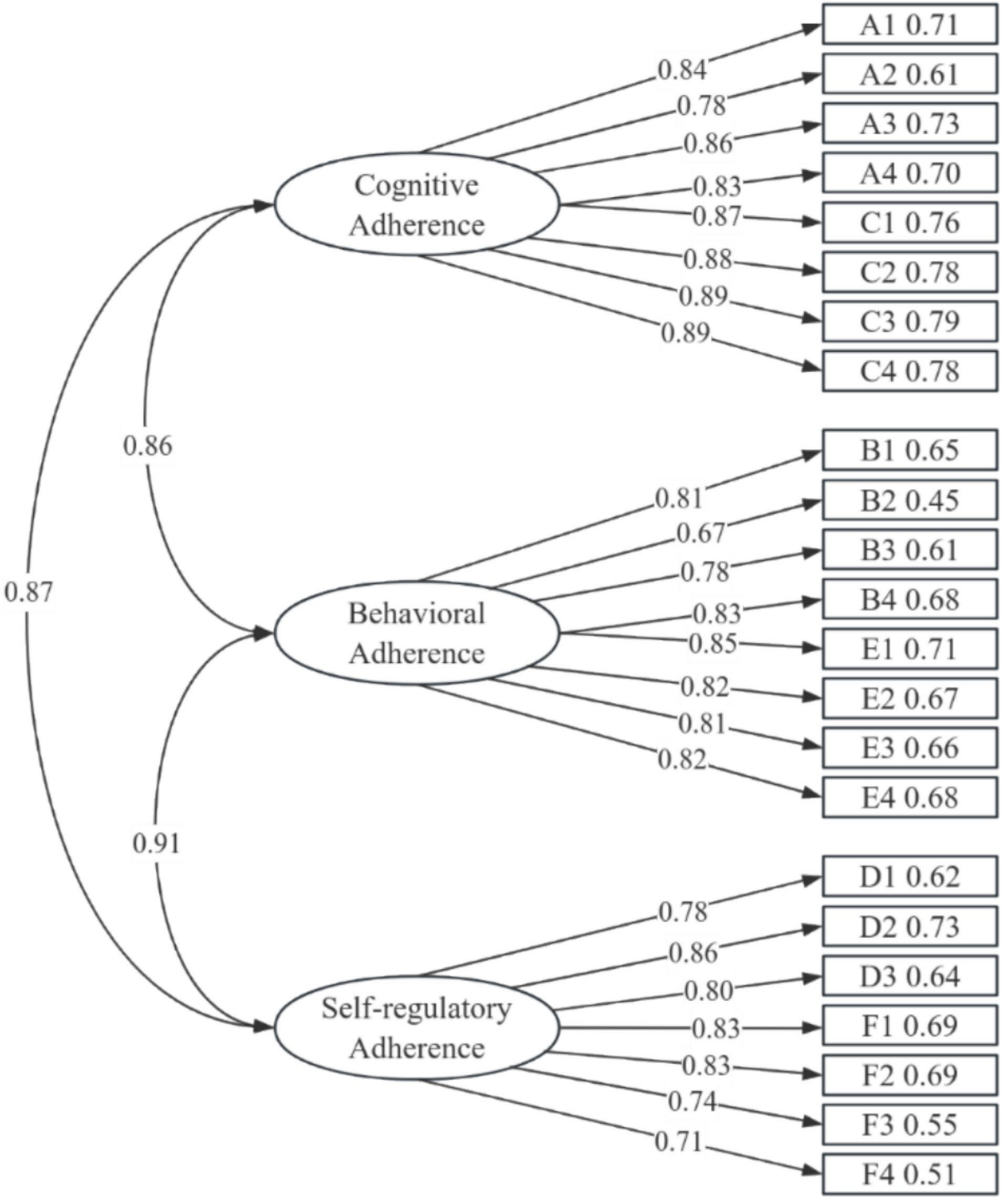
Factor structure model.

Using AMOS software, we constructed a structural equation model with the 24 items generated from exploratory factor analysis as observed variables and the three factors as latent variables, as shown in Figure 1.

To further verify the three-factor structure derived from exploratory factor analysis, a structural equation model was constructed by using AMOS software, with 23 items as observed variables and three factors as latent variables. In the structural equation model analysis process, after four modifications based on MI (Modification Index), the model parameters showed (Table 2) that although the χ2/df (chi-square/degrees of freedom) and RMSEA (root mean square error of approximation) indicators reached acceptable thresholds, other indicators such as NFI (normed fit index), CFI (comparative fit index), RMR (residual mean-square root), and GFI (goodness of fit index) all showed good fit. Overall, although some indicators did not reach the best fit level, they supported that the model fits the data well.

**Table 2:** Model fit results.

Fit index	Fit criteria	Test value	Test result
χ^2^/df	< 5, the model is acceptable < 3, model fits well	4.696	Acceptable
GFI	> 0.80, model fits well	0.80	Satisfactory
RMR	< 0.10, model is acceptable < 0.05, model fits well	0.05	Good
RMSEA	< 0.10, model is acceptable < 0.08, model fits well < 0.06, model fits very well < 0.01, model fits very perfectly	0.10	Acceptable
NFI	> 0.90, model fits well	0.87	Satisfactory
CFI	> 0.90, model fits well	0.88	Satisfactory

Further analysis of the standardized factor loadings of each item on the latent factors showed that all item loadings ranged from 0.51 to 0.78, all above the critical value of 0.50, and all reached the significance level (*p* < 0.001). These fit indicators comprehensively support the three-factor structure of ethnic traditional sports exercise prescription adherence. These three factors correspond to individual cognitive adherence, execution adherence, and self-regulatory adherence to exercise prescriptions. This finding is highly consistent with the behavioral change mechanisms emphasized by the Golden Six Principles of Exercise Prescription (FITT-VP), that is, an individual’s adherence behavior is the result of the combined effects of their subjective cognition of the prescription content, actual execution of the prescription, and self-monitoring and regulation during the execution process (Byrne, 2001). This multi-dimensional measurement of exercise adherence not only enriches and expands the theoretical connotations of exercise adherence but also provides important theoretical basis and empirical clues for the development of intervention strategies to improve adherence among the elderly.

Based on the results of the confirmatory factor analysis, we have formed an official scale consisting of three dimensions and 23 items. This official scale is divided into three sub-scales, with the total score and sub-scale scores calculated as “total score/number of corresponding items,” ranging from 1 to 5 points. The total score of the scale is referred to as the “level of adherence to ethnic traditional sports exercise prescriptions among the elderly”. The scores for each dimension and the total score are calculated by accumulation, with higher scores indicating a higher level of adherence to ethnic traditional sports exercise prescriptions.

### 3.4 Scale assessment

#### 3.4.1 Reliability testing

To examine the reliability level of the adherence scale for ethnic traditional sports exercise prescriptions, we employed two methods: internal consistency coefficient and split-half reliability. For internal consistency reliability, we calculated the Cronbach’s *α* coefficients for the total scale and each sub-scale. According to psychometric standards, an α coefficient of 0.90 or above for the total scale indicates excellent reliability; an α coefficient of 0.90 or above for sub-scales indicates high reliability (Devellis, 2016). In this study, the α coefficient for the total scale was 0.98, for the cognitive adherence sub-scale was 0.96, for the behavioral adherence sub-scale was 0.93, and for the self-regulation adherence sub-scale was 0.92, all reaching an ideal level. This indicates that the scale can measure the adherence levels of the elderly to ethnic traditional sports exercise prescriptions with high internal consistency, both as a whole and across different dimensions.

For split-half reliability, we used the odd–even method to calculate the Spearman–Brown coefficient. The results showed that the coefficient for the cognitive adherence sub-scale (A1, A2, A3, A4, C1, C2, C3, C4) was 0.91, for the behavioral adherence sub-scale (B1, B2, B3, B4, E1, E2, E3, E4) was 0.89, for the self-regulation adherence sub-scale (D1, D2, D3, F1, F2, F3, F4) was 0.87, and for the total scale was 0.93, all above the standard of 0.70, indicating very good split-half reliability.

The results of internal consistency and split-half reliability indicate that the scale has a very good level of reliability, which lays an important foundation for the subsequent application of the scale in research and practice. However, it should be pointed out that very good reliability does not fully guarantee the measurement quality of the scale, and further validity testing is needed to examine its measurement effectiveness.

#### 3.4.2 Validity testing

##### 3.4.2.1 Content validity

First, in the process of item development, we strictly followed the procedures of clarifying the conceptual scope, identifying key dimensions, and generating sample items, which were mainly derived from exercise prescription-related literature and expert interviews, ensuring that the measurement items comprehensively cover various aspects of adherence to ethnic traditional sports exercise prescriptions among the elderly. Second, when compiling the initial scale, we invited 20 elderly individuals to pilot the questionnaire and made repeated revisions based on their feedback, ensuring that the measurement items could clearly reflect the content of the concept under investigation. In summary, the measurement items of the adherence scale for ethnic traditional sports exercise prescriptions among the elderly are well-aligned with the target concept.

##### 3.4.2.2 Construct validity

Construct validity is used for situations where multiple indicators are measured, and it includes two subtypes: convergent validity and discriminant validity. Convergent validity refers to the tendency that items measuring the same construct will fall into the same factor. To test the convergent validity of the scale, the researchers calculated the average variance extracted (AVE) and CR for each factor. Table 3 shows that all factors have AVE values greater than the standard of 0.50, and all factors have CR values greater than 0.70, indicating that the scale has good convergent validity.

**Table 3:** Data on the scale’s convergent validity.

Factor	AVE	CR
Cognitive adherence	0.73	0.96
Behavioral adherence	0.64	0.93
Self-regulation adherence	0.63	0.92

Next, to test the scale’s discriminant validity, the square root of the AVE values was compared with the inter-factor Pearson correlation coefficients. Table 4 shows that the square root of the AVE values for the three factors is greater than the inter-factor correlation coefficients, indicating that the scale has good discriminant validity.

**Table 4:** Data on the scale’s discriminant validity.

Factor	Cognitive	Behavioral	Self-Regulation
Cognitive	0.86		
Behavioral	0.80	0.80	
Self-Regulation	0.78	0.79	0.80

##### 3.4.2.3 Criterion-related validity

Using the exercise adherence scale for patients with gastric cancer-related fatigue developed by Guizhen (2014) as the criterion, there was a significant positive correlation between adherence to ethnic traditional sports exercise prescriptions and adherence to exercise prescriptions for patients with gastric cancer-related fatigue (*r* = 0.39, *p* < 0.01), and all three dimensions of adherence to exercise prescriptions for patients with gastric cancer-related fatigue (exercise adherence, monitoring adherence, and seeking advice adherence) were significantly positively correlated (*r* = 0.36, *r* = 0.38, *r* = 0.38; *p* < 0.01). The three factors of adherence to ethnic traditional sports exercise prescriptions were also significantly correlated with the three factors of adherence to exercise prescriptions for patients with cancer-related fatigue, with correlation coefficients ranging from 0.25 to 0.60, *p* < 0.01, indicating good criterion-related validity of the scale.

In summary, the empirical data obtained from the adherence scale for ethnic traditional sports exercise prescriptions among the elderly are consistent with the inherent logic of the target concept. The results of the reliability and validity tests indicate that the scale has good quality and can be applied in practice.

## 4 Discussion

### 4.1 Conceptualization of adherence to ethnic traditional sports exercise prescriptions

Ethnic traditional sports refer to the totality of sports activities that have been passed down or inherited within different ethnic groups throughout history, primarily including the traditional disease prevention, fitness, martial arts, and recreational activities of various ethnic groups in China (Hong and Li, 2021). Based on a review of the literature, the medical field generally defines adherence as the consistency between an individual’s behavior and the recommendations of healthcare professionals, while the field of sports focuses on an individual’s participation in and continuous execution of a prescribed exercise plan (World Health Organization, 2003). These definitions often start from the perspective of medical professionals or researchers, neglecting the subjective experience of the elderly and not fully considering the specific context of ethnic traditional sports exercise prescriptions. Therefore, this study aims to redefine adherence to ethnic traditional sports exercise prescriptions from the perspective of the elderly’s intrinsic experience, which refers to the cognitive, behavioral, and self-regulatory tendencies formed by individuals during their participation in ethnic traditional sports, reflecting the degree of consistency between individual behavior and exercise recommendations. This concept includes not only the identification and internalization at the cognitive and behavioral levels but also the consistency in self-regulation to meet the requirements of the exercise prescription. From the perspective of the Golden Six Principles of Exercise Prescription (FITT-VP), adherence to ethnic traditional sports exercise prescriptions is mainly influenced by the elderly’s cognition, behavior, and self-regulation. For example, an individual’s understanding of the basic elements of ethnic traditional sports exercise prescriptions can affect the performance of their adherence behavior.

### 4.2 Structural dimensions of adherence to ethnic traditional sports exercise prescriptions

Based on previous discussions of the survey questionnaire and in line with the Golden Six Principles of Exercise Prescription (FITT-VP), this study has developed an adherence scale for ethnic traditional sports exercise prescriptions, which includes 23 items across three dimensions. This scale takes into full consideration the unique experiences of the elderly in China’s cultural context when participating in traditional ethnic sports and reflects the connotations of adherence to these exercise prescriptions (Morris, 2004). Specifically, the cognitive dimension focuses on the elderly’s memory, understanding, and beliefs about the content of the exercise prescription, the behavioral dimension highlights the actual implementation of the exercise prescription, and the self-regulation dimension reflects the elderly’s ability to perceive, record, and adjust to changes in exercise types and progression during their workout process (Hong and Li, 2021). These three dimensions are relatively independent yet closely linked, together constituting the complete connotation of adherence to ethnic traditional sports exercise prescriptions and embodying the interactive effects among personal cognition, behavioral performance, and self-regulatory abilities emphasized by the Golden Six Principles of Exercise Prescription (FITT-VP).

During the development process, based on literature reviews, researchers collected real feelings of the elderly participating in traditional ethnic sports through semi-structured interviews and invited experts in the fields of sports science, psychology, and geriatric medicine to review and revise the items, ensuring the content validity of the scale (Lynn, 1986). Preliminary survey results showed that the scale’s language is easy to understand and convenient to fill out, with a high acceptance rate among the elderly. The results of the confirmatory factor analysis further support the three-factor structure of the scale, indicating that the division of dimensions is reasonable and the factor structure is stable (Tabachnick and Fidell, 2019). The analysis of reliability and validity indicates that the scale meets the standards of psychometrics for internal consistency, test–retest reliability, structural validity, and criterion-related validity.

In summary, the adherence scale for ethnic traditional sports exercise prescriptions developed in this study has high quality in terms of theoretical foundation, content development, and reliability and validity testing (Kyriazos, 2018). It can effectively assess the multi-dimensional adherence of the elderly to ethnic traditional sports exercise prescriptions, providing a reliable measurement tool for subsequent large-sample survey research and the development of strategies to improve adherence among the elderly (Schumacker and Lomax, 2012). At the same time, the scale closely follows the characteristics of ethnic traditional sports and highlights the perspective of Chinese local culture, also providing new empirical support for enriching the theoretical connotations of exercise adherence.

### 4.3 Applicability of the scale and future research directions

This study has sample limitations, as it only included retired university teachers from Guangdong province, which may restrict the generalizability of results to the broader elderly population. While this relatively homogeneous sample is beneficial for initial scale development, it presents certain limitations in terms of representativeness. However, our meticulous individual data collection approach, though resulting in a smaller sample size, ensured data authenticity and reliability, accurately reflecting the actual exercise prescription adherence patterns among elderly individuals. After being tested with Cronbach’s *α* coefficient and confirmatory factor analysis, the adherence scale for ethnic traditional sports exercise prescriptions has achieved ideal levels of reliability for each dimension and the total scale. The validity of the scale, tested through content and structural validity (exploratory and confirmatory factor analysis), also shows satisfactory structural stability of the three-factor model (Nunnally and Bernstein, 1994). Therefore, it is concluded that this scale can serve as an effective assessment tool for the middle-aged and elderly population in China. This result is beneficial to some extent for promoting in-depth research on adherence to ethnic traditional sports exercise prescriptions. At the same time, to further refine the structure and content of the scale and to make it more widely applicable, it is suggested that future researches continue to test and verify its reliability and validity, as well as the stability of its factor structure, with larger samples of the elderly in different regions (Fabrigar et al., 1999). To further refine the scale and enhance its applicability, future research should validate its reliability, validity, and factor structure with larger elderly samples from diverse regions and occupational backgrounds. We plan to expand our sample range to include more diverse elderly populations while maintaining rigorous data collection methods to ensure data authenticity and reliability. This expansion is essential for establishing the scale’s universal applicability and will provide a more solid foundation for research on exercise prescription adherence among the elderly. Based on the three core dimensions of adherence to ethnic traditional sports exercise prescriptions revealed by this scale, it is recommended that when developing exercise intervention strategies for the elderly, the following factors should be fully considered: First, at the cognitive level, it is recommended to strengthen the elderly’s understanding of the content and benefits of exercise prescriptions to enhance their exercise beliefs and determination; second, at the behavioral level, it is suggested to provide the elderly with specific and feasible exercise guidance and to create a convenient exercise environment to reduce exercise barriers; finally, at the self-regulation level, it is advised to guide the elderly to learn to identify exercise types, judge their own exercise progression stages, adjust their exercise plans, and provide feedback to coaches or exercise prescription specialists in a timely manner to enhance their sense of self-regulation in exercise. In summary, adopting targeted strategies from different dimensions during the implementation of exercise prescriptions can effectively improve the exercise adherence levels of the elderly.

## 5 Conclusion

This study developed and validated a novel scale to measure adherence to ethnic traditional sports exercise prescriptions among older adults, integrating the Golden Six Principles of Exercise Prescription (FITT-VP) with Social Cognitive Theory. Through literature review, expert consultation, semi-structured interviews, and rigorous psychometric testing—including item analysis, exploratory factor analysis, confirmatory factor analysis, and reliability and validity evaluations—the resulting 23-item, three-dimensional structure (Cognitive, Behavioral, and Self-Regulatory Adherence) demonstrated robust internal consistency, satisfactory test–retest reliability, and strong evidence of construct and criterion-related validity. The scale not only provides a valuable tool for accurately assessing the adherence levels of older adults engaging in ethnic traditional sports but also underscores the importance of considering cultural factors, self-regulatory processes, and comprehensive exercise prescription principles. These findings lay a theoretical and empirical foundation for future large-scale surveys and targeted interventions to improve adherence to ethnic traditional sports prescriptions, ultimately contributing to healthy aging and chronic disease prevention in the elderly population.

## Data Availability

The raw data supporting the conclusions of this article will be made available by the authors, without undue reservation.

## References

[ref1] Al S. (1998). Patterns of exercise and fatigue in physically active cancer survivors. PubMed 25, 485–491. 9568604

[ref2] Bandura A. (2004). Health promotion by social cognitive means. Health Educ. Behav. 31, 143–164. doi: 10.1177/1090198104263660, PMID: 15090118

[ref3] Bauman A. E. Reis R. S. Sallis J. F. Wells J. C. Loos R. J. Martin B. W. (2012). Correlates of physical activity: why are some people physically active and others not? Lancet 380, 258–271. doi: 10.1016/s0140-6736(12)60735-1, PMID: 22818938

[ref4] Byrne B. M. (2001). Structural equation modeling with AMOS: Psychology Press.

[ref5] Chen H.-H. Yeh M.-L. Lee F.-Y. (2006). The effects of Baduanjin qigong in the prevention of bone loss for middle-aged women. Am. J. Chin. Med. 34, 741–747. doi: 10.1142/s0192415x06004259, PMID: 17080541

[ref6] Devellis R. F. (2016). Scale development: Theory and applications. Thousand Oaks, CA: Sage Publications, Inc.

[ref8] Ekkekakis P. Parfitt G. Petruzzello S. J. (2011). The pleasure and displeasure people feel when they exercise at different intensities. Sports Med. 41, 641–671. doi: 10.2165/11590680-000000000-00000, PMID: 21780850

[ref9] Fabrigar L. R. Wegener D. T. MacCallum R. C. Strahan E. J. (1999). Evaluating the use of exploratory factor analysis in psychological research. Psychol. Methods 4, 272–299. doi: 10.1037/1082-989x.4.3.272

[ref10] Fetherston C. M. Wei L. (2011). The benefits of tai chi as a self management strategy to improve health in people with chronic conditions. J. Nurs. Healthc. Chronic Illn. 3, 155–164. doi: 10.1111/j.1752-9824.2011.01089.x

[ref11] Field A. P. (2018). Discovering statistics using IBM SPSS statistics. 5th Edn. London, UK: Sage.

[ref260] Guizhen W. (2014). A Research on the Gastric Cancer-related Fatigue Patients’ Compliance to Exercise Prescription. Fujian Medical University.

[ref12] Hong F. Li L. (2021). Indigenous sports history and culture in Asia. London, UK: Routledge.

[ref13] Kyriazos T. A. (2018). Applied psychometrics: sample size and sample power considerations in factor analysis (EFA, CFA) and SEM in general. Psychology 9, 2207–2230. doi: 10.4236/psych.2018.98126

[ref14] Lynn M. R. (1986). Determination and quantification of content validity. Nurs. Res. 35, 382–386. doi: 10.1097/00006199-198611000-00017 3640358

[ref15] Morris A. D. (2004). Marrow of the nation: A history of sport and physical culture in republican China. Berkeley & Los Angeles, CA: University Of California Press.

[ref16] Nunnally J. C. Bernstein I. H. (1994). Psychometric theory (3rd ed.). New York, NY: McGraw-Hill.

[ref17] Pinto B. M. Rabin C. Dunsiger S. (2009). Home-based exercise among cancer survivors: adherence and its predictors. Psycho-Oncology 18, 369–376. doi: 10.1002/pon.1465, PMID: 19242921 PMC2958525

[ref18] Polit D. F. Beck C. T. (2006). The content validity index: are you sure you know what’s being reported? Critique and recommendations. Res. Nurs. Health 29, 489–497. doi: 10.1002/nur.20147, PMID: 16977646

[ref19] Rhodes R. E. Janssen I. Bredin S. S. D. Warburton D. E. R. Bauman A. (2017). Physical activity: health impact, prevalence, correlates and interventions. Psychol. Health 32, 942–975. doi: 10.1080/08870446.2017.1325486, PMID: 28554222

[ref20] Sallis J. F. Floyd M. F. Rodríguez D. A. Saelens B. E. (2021). Role of built environments in physical activity, obesity, and cardiovascular disease. Circulation 125, 729–737. doi: 10.1161/circulationaha.110.969022 PMC3315587 22311885

[ref21] Schumacker R. E. Lomax R. G. (2012). A beginner’s guide to structural equation modeling (3rd ed.). New York, NY: Taylor & Francis.

[ref22] Sniehotta F. F. Scholz U. Schwarzer R. (2005). Bridging the intention–behaviour gap: planning, self-efficacy, and action control in the adoption and maintenance of physical exercise. Psychol. Health 20, 143–160. doi: 10.1080/08870440512331317670

[ref23] Tabachnick B. G. Fidell L. S. (2019). Using multivariate statistics (7th ed.). Boston Pearson.

[ref24] Waltz C. F. Bausell R. B. (1981). Nursing research. Philadelphia, PA: F A Davis Company.

[ref25] Warburton D. E. R. Bredin S. S. D. (2017). Health benefits of physical activity: a systematic review of current systematic reviews. Curr. Opin. Cardiol. 32, 541–556. doi: 10.1097/hco.0000000000000437, PMID: 28708630

[ref26] World Health Organization (2003). Adherence to long-term therapies: Evidence for action. Geneva: World Health Organization.

[ref27] Yeh G. Y. Wood M. J. Lorell B. H. Stevenson L. W. Eisenberg D. M. Wayne P. M.. (2004). Effects of tai chi mind-body movement therapy on functional status and exercise capacity in patients with chronic heart failure: a randomized controlled trial. Am. J. Med. 117, 541–548. doi: 10.1016/j.amjmed.2004.04.016, PMID: 15465501

